# Choroid plexus volume in multiple sclerosis can be estimated on structural MRI avoiding contrast injection

**DOI:** 10.1186/s41747-024-00421-9

**Published:** 2024-02-27

**Authors:** Valentina Visani, Francesca B. Pizzini, Valerio Natale, Agnese Tamanti, Mariagiulia Anglani, Alessandra Bertoldo, Massimiliano Calabrese, Marco Castellaro

**Affiliations:** 1 Department of Information Engineering, University of Padova, Padova, Italy; 2 Department of Engineering for Innovation Medicine, University of Verona, Verona, Italy; 3 Department of Diagnostic and Public Health, University of Verona, Verona, Italy; 4 Department of Neurosciences, Biomedicine and Movement Sciences, University of Verona, Verona, Italy; 5 Neuroradiology Unit, University Hospital of Padova, Padova, Italy; 6 Padova Neuroscience Center, University of Padova, Padova, Italy

**Keywords:** Choroid plexus, Gadolinium-based contrast media, Image segmentation, Magnetic resonance imaging, Multiple sclerosis

## Abstract

We compared choroid plexus (ChP) manual segmentation on non-contrast-enhanced (non-CE) sequences and reference standard CE T1- weighted (T1w) sequences in 61 multiple sclerosis patients prospectively included. ChP was separately segmented on T1w, T2-weighted (T2w) fluid-attenuated inversion-recovery (FLAIR), and CE-T1w sequences. Inter-rater variability assessed on 10 subjects showed high reproducibility between sequences measured by intraclass correlation coefficient (T1w 0.93, FLAIR 0.93, CE-T1w 0.99). CE-T1w showed higher signal-to-noise ratio and contrast-to-noise ratio (CE-T1w 23.77 and 18.49, T1w 13.73 and 7.44, FLAIR 13.09 and 10.77, respectively). Manual segmentation of ChP resulted 3.073 ± 0.563 mL (mean ± standard deviation) on T1w, 3.787 ± 0.679 mL on FLAIR, and 2.984 ± 0.506 mL on CE-T1w images, with an error of 28.02 ± 19.02% for FLAIR and 3.52 ± 12.61% for T1w. FLAIR overestimated ChP volume compared to CE-T1w (*p* < 0.001). The Dice similarity coefficient of CE-T1w *versus* T1w and FLAIR was 0.67 ± 0.05 and 0.68 ± 0.05, respectively. Spatial error distribution per slice was calculated after nonlinear coregistration to the standard MNI152 space and showed a heterogeneous profile along the ChP especially near the fornix and the hippocampus. Quantitative analyses suggest T1w as a surrogate of CE-T1w to estimate ChP volume.

**Relevance statement** To estimate the ChP volume, CE-T1w can be replaced by non-CE T1w sequences because the error is acceptable, while FLAIR overestimates the ChP volume. This encourages the development of automatic tools for ChP segmentation, also improving the understanding of the role of the ChP volume in multiple sclerosis, promoting longitudinal studies.

**Key points** • CE-T1w sequences are considered the reference standard for ChP manual segmentation.

• FLAIR sequences showed a higher CNR than T1w sequences but overestimated the ChP volume.

• Non-CE T1w sequences can be a surrogate of CE-T1w sequences for manual segmentation of ChP.

**Graphical Abstract:**

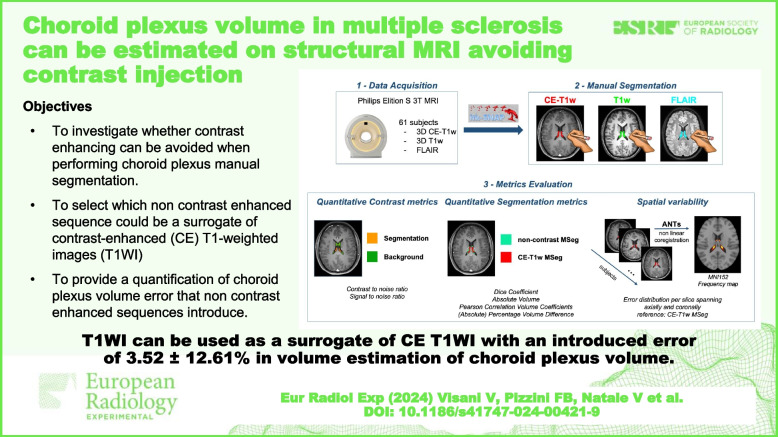

## Background

The choroid plexus (ChP) is a vascular tissue located in the brain ventricular system in the four ventricles, and it forms a major part of the barrier between blood and cerebrospinal fluid. The main role of the ChP is the production of the majority of the cerebrospinal fluid [1]. Moreover, ChP is a mediator of the brain clearance pathways that allow maintaining brain homeostasis [2, 3]. Consequently, it can be considered part of the glymphatic system [4]. In addition, the ChP is involved in inflammatory processes, and it has been suggested to further investigate ChP role in promoting intrathecal inflammation mechanisms [5]. Therefore, the functional and anatomical modification of the ChP can lead to alterations that help characterize neurodegenerative pathologies like multiple sclerosis [5–9], Alzheimer’s disease [2, 10, 11], or psychiatric disorders [12–14]. The functions of the ChP have been investigated using different quantitative imaging modalities, like magnetic diffusion-weighted [15] and perfusion [16] magnetic resonance imaging (MRI), or positron emission tomography using [^11^C](R)PK11195, a marker of activated microglia [12], and [^11^C]PIB, which detect amyloid load [17]. ChP alteration can be also quantified by calculating its volume, which has been found severely enlarged in neurological disorders [7, 8, 12, 14]. A recent study [18] has hypothesized a correlation between the inflammatory state, the ChP volume, and the multiple sclerosis stage, proposing the ChP volume as a possible biomarker to better understand the evolution of the disease [19].

The high contrast and resolution of structural MRI have made it the natural choice to perform ChP manual segmentation (MSeg). In fact, the ChP imaging reference standard technique is contrast-enhanced (CE) T1weighted (T1w) sequences, *i.e.*, after intravenous administration of gadolinium-based contrast agent [10, 20]. This approach is somehow more invasive than using simple non-CE T1w sequences [21], but its utility remains unquestioned, and therefore, it is routinely acquired in multiple sclerosis initial diagnostic procedures, whereas its use is controversial in follow-up [22]. To the best of our knowledge, despite the previously reported use of non-CE T1w sequences for ChP MSeg [7], a quantitative assessment of the performance of ChP MSeg on non-CE sequences with respect to the reference standard given by CE-T1w on a large cohort of patients has never been done.

**Fig. 1:**
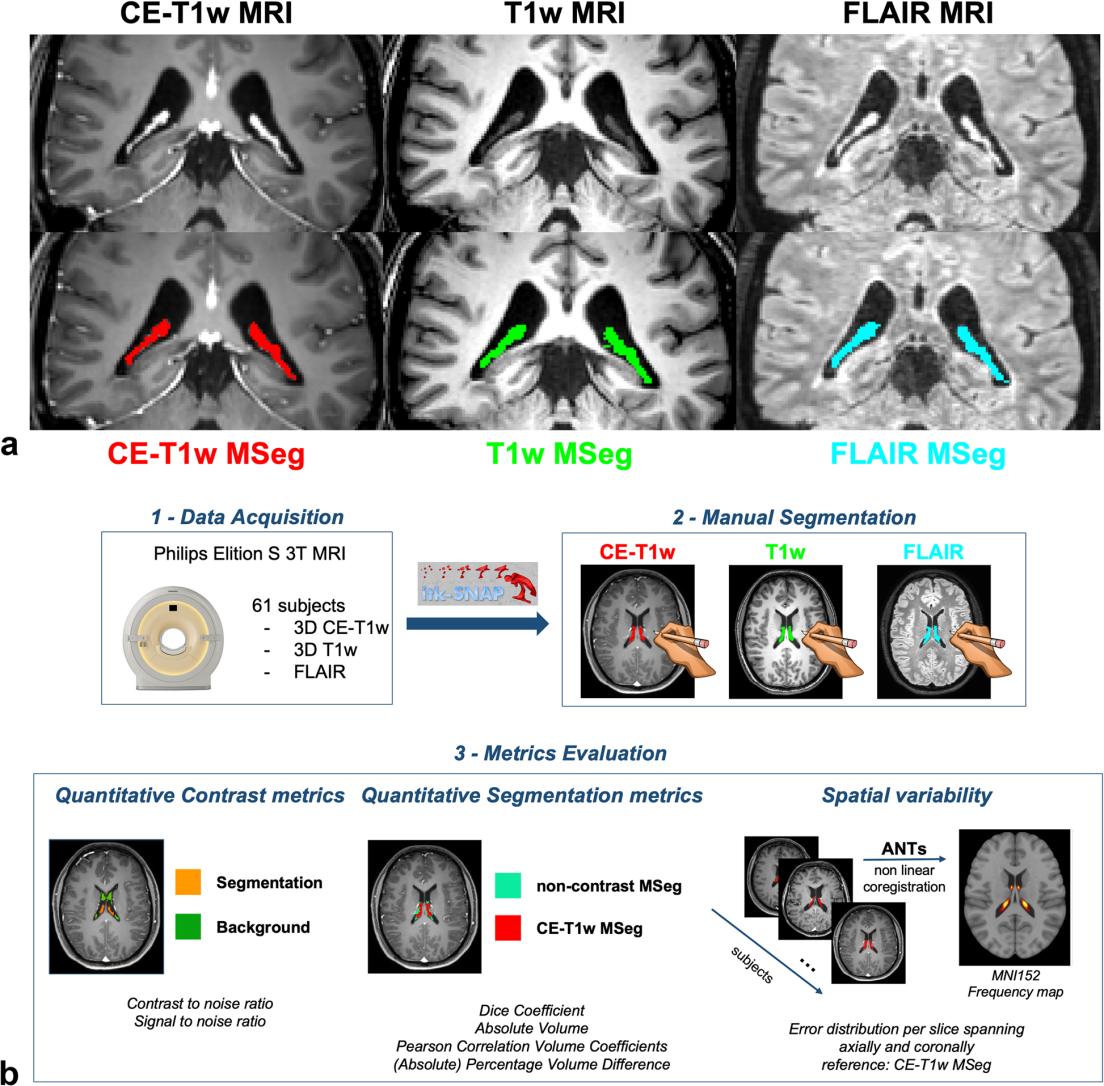
**a** Coronal view of choroid plexus of a representative patient. First row, from left to right: contrast-enhanced (CE) T1-weighted (T1w), non-CE T1w, and fluid-attenuated inversion-recovery (FLAIR) sequences. Second row, from left to right; previous images with overlapped, respectively, CE-T1w manual segmentation (MSeg) in red, T1w MSeg in green, and FLAIR MSeg in blue. **b** Study workflow: (1) data acquisition (for details, see the “Data acquisition” section); (2) MSeg performed by neuroradiologists on ITK-snap for each available sequence for each subject (for details, see the “Manual segmentation and inter-rater agreement” section); (3) metrics evaluation: analyses performed in the study (for details, see the “Quantitative contrast metrics,” “Quantitative segmentation metrics,” and “Spatial variability” sections). *ANTs*, Advanced Normalization Toolbox

The aim of this work was to compare the ChP segmentations manually depicted on T1w, T2-weighted fluid-attenuated inversion-recovery, and CE-T1w sequences (Fig. 1a) to determine whether non-CE sequences are accurate enough to be used in quantitative studies of ChP volume. In fact, CE-T1w sequences are not routinely acquired for all neurological settings; thus, several studies employed commonly available unenhanced T1w sequences to quantitatively estimate the ChP volume [23].

## Methods

The study workflow is shown in Fig. 1b and was composed by the following steps.

### Data acquisition

All patients gave their written informed consent prior to participating in the study. All procedures were performed in accordance with the Declaration of Helsinki (2008), and the study protocol was approved by the local ethical committee. Sixty-one relapsing-remitting multiple sclerosis patients (aged 39.9 ± 9.5 years, mean ± standard deviation) acquired prospectively between November 2020 and October 2021 were included. Images were acquired on a Elition-S 3T scanner (Philips Healthcare, Best, The Netherlands), equipped with a 32-channel head coil, using this protocol:

i. Three-dimensional T1w magnetization-prepared and gradient-echo, MPRAGE (compressed sensing sensitivity encoding factor 4; echo time/repetition time 3.8/8.5 ms; flip angle 8°; voxel size 1 × 1 × 1 mm^3^);
ii. Three-dimensional T2w turbo spin-echo FLAIR (compressed sensing sensitivity encoding factor 5; echo time/repetition time 376/8,000 ms; inversion time 2,356 ms; voxel size 1 × 1 × 1 mm^3^);
iii. CE-T1w same sequence as in point (i) after 10 min from intravenous injection of contrast agent (gadobutrol, Gadovist®, Bayer AG, Leverkusen, Germany) at the dose of 0.1 mmol/kg of bodyweight with an injection rate of 2 mL/s.

### Manual segmentation and inter-rater agreement

The manual segmentation procedure was done in a common space on CE-T1w and FLAIR after coregistering using an affine transformation of both sequences to the T1w sequence. A junior neuroradiologist (V.N., 2-year experience) depicted the MSegs of the ChP in the two lateral ventricles [23] on each sequence and for each patient using ITK-snap [24], and a senior neuroradiologist (F.B.P, 18-year experience) confirmed the segmentations, editing them in 20% of cases. Patients were randomized, and each sequence was segmented separately. The order of segmentation was T1w, FLAIR, and CE-T1w to limit the rater bias towards the CE-T1w sequence.

A second expert neuroradiologist (M.G.A., 9-year experience) replicated the manual segmentation procedure to test the inter-rater variability of each sequence on ten subjects chosen randomly from the 61 available. Before starting the inter-rater evaluation, three representative subjects already segmented from the first team were provided as examples to the second neuroradiologist to standardize the segmentation procedure. Inter-rater agreement and similarity of the three available sequences was performed comparing each operator segmentation mask. The Dice similarity coefficient (DSC) was used to test the spatial overlap, whereas the intraclass correlation coefficient (ICC) on the absolute volume was calculated to evaluate the overall inter-rater agreement between operators.

### Quantitative contrast metrics

We investigated the contrast-to-noise ratio (CNR) and the signal-to-noise ratio (SNR) between the ChP and the background region (lateral ventricles). The ChP region-of-interest was obtained from the MSeg. The background region-of-interest was obtained as follows: first, a raw ventricles segmentation was extracted from the FreeSurfer (v7.1.1) pipeline [25]; second, the raw ventricle mask was refined by excluding the union of the ChP masks obtained for each sequence (T1w, FLAIR, and CE-T1w); third, an erosion operation (spherical kernel of 2 mm) was applied to include only background voxels.

### Quantitative segmentation metrics

To quantitatively investigate the differences between the MSegs obtained from the available sequences, we calculated for each subject, using the cT1w MSeg as reference: the DSC, the absolute volume, the Pearson correlation volume coefficients, the percentage volume difference (ΔVol%), the absolute percentage volume difference (|ΔVol%|).

### Spatial variability

To assess the spatial variability of each sequence, we nonlinearly coregistered each subject, using the Advanced Normalization Toolbox [26], to the MNI Talairach ICBM 152 2009c Nonlinear Symmetric template (MNI152) [27]. We evaluated the error distribution per slice spanning axially and coronally to provide quantitative metrics of agreement between the non-contrast-enhanced sequences and the CE-T1w sequence. Lastly, we constructed the frequency maps reporting for each voxel the probability of being ChP for each MSegs.

### Statistics

Inter-rater agreement was statistically evaluated using one-way ANOVA and post hoc *t*-test (significance level *α* = 0.05) between the DSC among the available sequences. One-way ANOVA and post hoc *t*-test (significance level *α* = 0.05) between the available sequences were performed for both SNR and CNR values. Regarding quantitative segmentation metrics, one-way ANOVA and post hoc *t*-test (*α* = 0.05) were performed for absolute volume metric between all available MSegs. One-sample *t*-test (*α* = 0.05) was performed for ΔVol% metric for both non-CE MSegs. Two-sample *t*-test (*α* = 0.05) was performed for both ΔVol% and |ΔVol%| metrics between the two non-CE MSegs.

## Results

The inter-rater analysis overlap using the DSC reported a high degree of overlap between operators (T1w 0.90 ± 0.02, FLAIR 0.88 ± 0.04, CE-T1w 0.87 ±0.06). ANOVA did not show any statistically significant difference in DSC between sequences (*p* = 0.304). Likewise, the agreement, investigated with the absolute volume ICC, shows very consistent reproducibility between operators, with CE-T1w showing the higher agreement (T1w 0.93, FLAIR 0.93, CE-T1w 0.99).

**Fig. 2:**
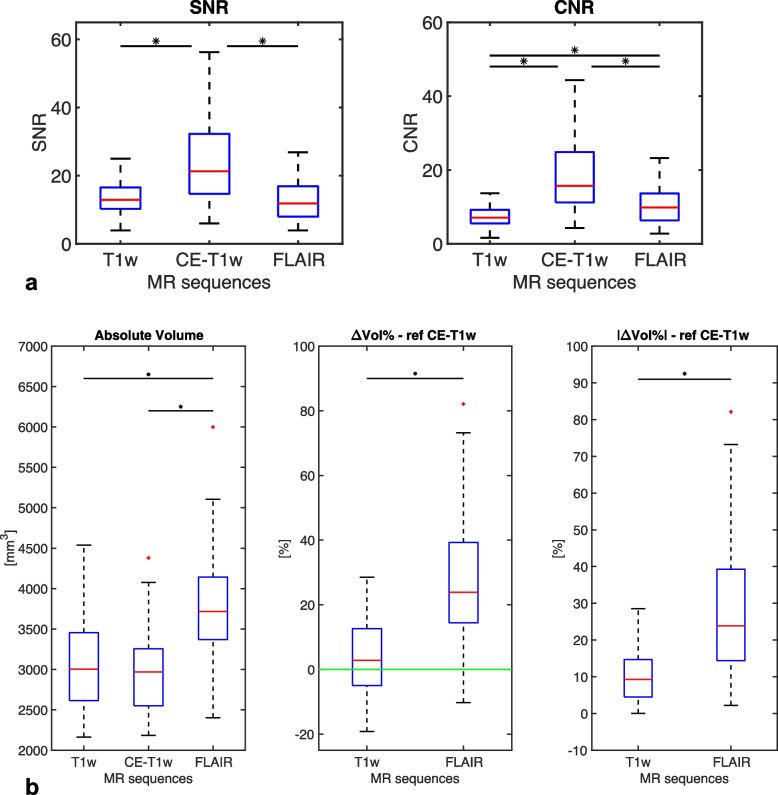
**a** Quantitative contrast metrics. Boxplot of signal-to-noise ratio (SNR) and contrast-to-noise ratio (CNR) between the choroid plexus and the ventricles for the three compared sequences: T1-weighted (T1w), fluid-attenuated inversion-recovery (FLAIR), and contrast-enhanced (CE) T1w. The asterisk indicates that the metrics between the two groups are statistically different (*p* < 0.05). **b** Quantitative segmentation metrics, from left to right: boxplot of the absolute volume of the manual segmentations (MSegs); boxplot of the percentage volume difference (ΔVol%) for T1w and FLAIR MSegs (reference CE-T1w); boxplot of the absolute percentage volume difference (|ΔVol%|) for T1w and FLAIR MSegs (reference CE-T1w). The asterisk indicates statistically significant differences between groups (*p* < 0.05)

Table 1 reports the results for both the quantitative contrast and segmentation metrics. For both the contrast metrics (SNR and CNR), the ANOVA revealed a statistically significant main effect between the tested sequences (*p* < 0.001 for both SNR and CNR). As shown in Fig. 2a, the post hoc *t*-tests between each sequence combination showed a significantly higher SNR and CNR for CE-T1w when compared to both T1w and FLAIR: SNR and CNR were 23.77 and 18.49 for CE-T1w, 13.73 and 7.44 for T1w, and 13.09 and 10.77 for FLAIR. FLAIR showed a significantly higher CNR when compared to T1w (*p* < 0.001). *t*-test between T1w and FLAIR sequences for SNR did not show significant differences (*p* = 0.103).

**Table 1:** Results and formulations of quantitative contrast metrics and quantitative segmentation metrics

Metrics	T1w	CE-T1w	FLAIR
***Quantitative contrast metrics***			
*Signal-to-noise ratio* $\frac{mean\left( RO{I}_{values}\right)}{std\left( backgroun{d}_{values}\right)}$	13.73 ± 4.89	23.77 ± 12.02	13.09 ± 6.15
*Contrast-to-noise ratio* $\frac{mean\left( RO{I}_{values}\right)- mean\left( backgroun{d}_{values}\right)}{std\left( backgroun{d}_{values}\right)}$	7.44 ± 2.87	18.49 ± 9.57	10.77 ± 5.38
***Quantitative segmentation metrics***			
*Dice similarity coefficient* $\frac{2\ast TP}{2\ast TP+ FP+ FN}$	0.67 ± 0.05	-	0.68 ± 0.05
*Absolute volume [mL]*	3.073 ± 0.563 mL	2.984 ± 0.506 mL	3.787 ± 0.679 mL
*Percentage volume difference* $100\ast \frac{\left( Volum{e}_{segm}- Volum{e}_{ref}\right)}{Volum{e}_{ref}}\left[\%\right]$	3.52 ± 12.61	-	28.02 ± 19.02
*Absolute percentage* *volume difference* $100\ast \frac{\mid Volum{e}_{segm}- Volum{e}_{ref}\mid }{Volum{e}_{ref}}\left[\%\right]$	10.57 ± 7.60	-	28.43 ± 18.40
*Pearson’ correlation*	0.77	-	0.67
***Spatial variability metrics***			
*Error distribution per slice*	$100\ast \frac{\left( VoxelSlice{(i)}_{segm}- VoxelSlice{(i)}_{ref}\right)}{Volum{e}_{ref}}\left[\%\right]$		

Data are presented as mean ± standard deviation. Quantitative contrast metrics: contrast-to-noise ratio and signal-to-noise ratio between choroid plexus (ChP) manual segmentation (MSeg) obtained from each sequence and a reference region obtained excluding the ChP from the ventricles (for details, see the “Quantitative contrast metrics” section). Quantitative segmentation metrics: Dice similarity coefficient, absolute volume, absolute percentage volume difference, and Pearson volume correlation analysis between MSegs obtained from non-contrast enhanced sequences (T1w and FLAIR MSeg) and the reference contrast-enhanced T1-weighted MSeg. *FN*, False negative; *FP*, False positive; *TP*, True positive

The pair-wise correlation analysis between each MSeg obtained from each sequence reported a significant positive correlation between the ChP volume for CE-T1w MSeg and both non-CE sequences (T1w *versus* CE-T1w 0.77; FLAIR *versus* CE-T1w: 0.67). One-way ANOVA revealed a significant main effect between tested MSegs (*p* < 0.001). FLAIR provided a higher absolute volume when compared to both T1w and CE-T1w MSegs (*p* < 0.001 for both), while there were no significant differences between T1w and CE-T1w MSegs volumes (*p* = 0.362). T1w MSeg provides lower |ΔVol%| (10.57 ± 7.60%, mean ± standard deviation) compared to FLAIR MSeg (28.43 ± 18.40%), which overestimated the ChP volume for every subject (ΔVol% for T1w 3.52 ± 12.61%; ΔVol% for FLAIR 28.02 ± 19.02%) (Fig. 2b). Statistical tests on |ΔVol%| and ΔVol% confirmed significant differences between the two non-CE MSegs (*p* < 0.001 for both). One-sample *t*-test on ΔVol% revealed T1w was normally distributed with non-zero mean (*p* = 0.033). The MSegs obtained from non-CE sequences showed similar DSCs (T1w *versus* CE-T1w 0.67; FLAIR *versus* CE-T1w 0.68) (see Table 1).

**Fig. 3:**
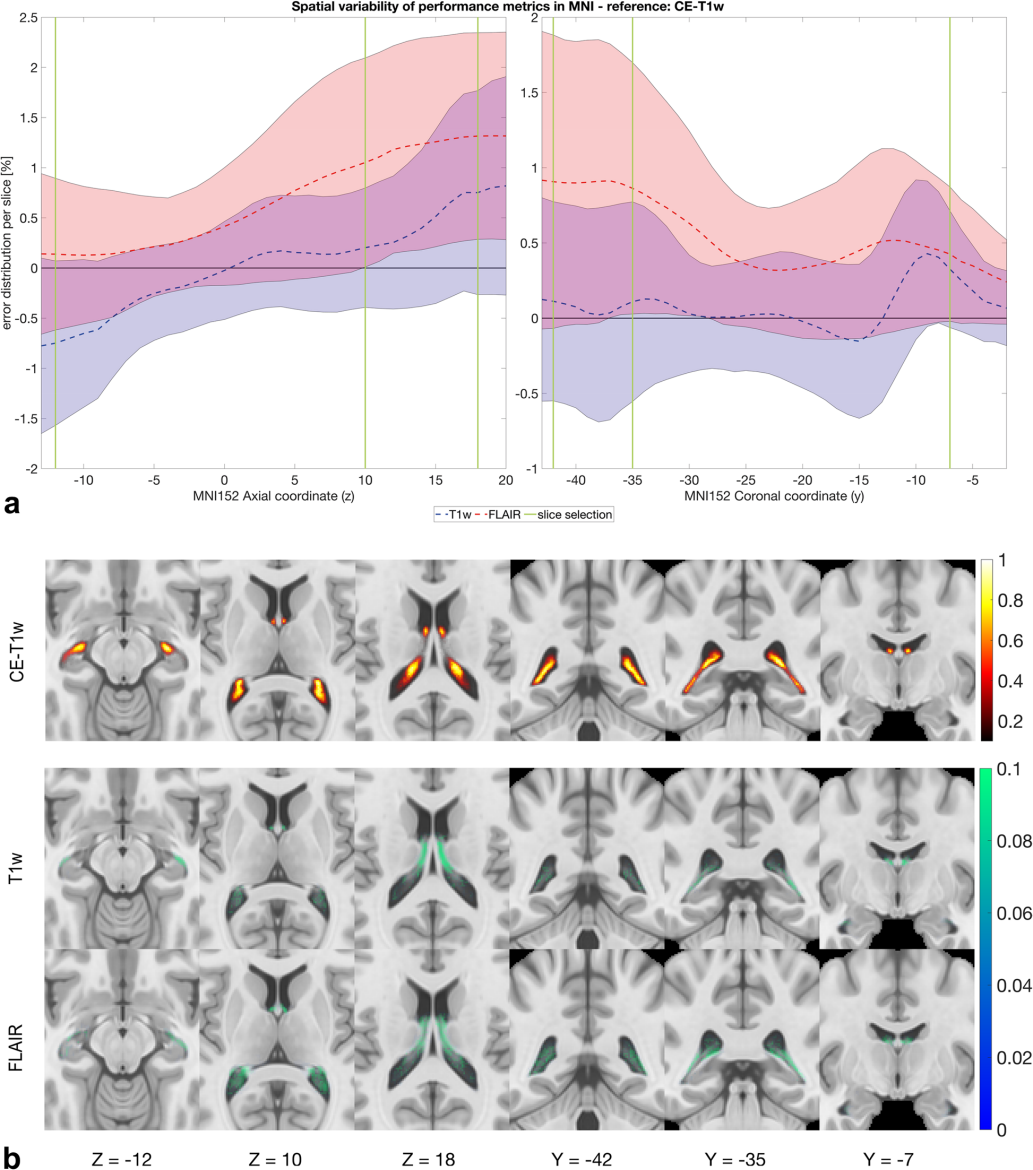
**a** Slice-wise evaluation of performance metrics in MNI Talairach ICBM 152 2009c nonlinear symmetric template (MNI152) coordinate system. The graph represents the error distribution per slice between non-contrast-enhanced (CE) manual segmentations (MSegs)—fluid-attenuated inversion-recovery (FLAIR) in red, T1-weighted (T1w) in blue—and the reference CE-T1w computed axial-wise along the *z*-coordinate (on the left) and coronal-wise along the *y*-coordinate (on the right) of the MNI152 coordinate system. The vertical green lines highlight three representative axial (*z* = -12: temporal horn near the head of the hippocampus; *z* = 10: atrium of the lateral ventricle; *z* = 18: body of the lateral ventricles near the fornix) and coronal (*y* = -35: anterior portion of the atrium of the lateral ventricles; *y* = -42: posterior portion of the atrium of the lateral ventricles; *y* = -7: anterior portion of the fornix) slices reported in **b**. **b** Probability frequency maps of MSegs overlapped to the MNI Talairach ICBM 152 2009c nonlinear symmetric template (MNI152). From left to right, three axial views (*z* = -12: temporal horn near the head of the hippocampus; *z* = 10: atrium of the lateral ventricle; *z* = 18: body of the lateral ventricles near the fornix) and three coronal views (*y* = -35: anterior portion of the atrium of the lateral ventricles; *y* = -42: posterior portion of the atrium of the lateral ventricles; *y* = -7: anterior portion of the fornix). Reported coordinates are centered at the origin of the MNI space. The first row represents the frequency map of the CE-T1w MSeg for representative slices overlapped to the MNI152 template. The second and third rows show the difference between the frequency map of MSegs depicted on non-CE sequences (T1w and FLAIR) and the MSeg obtained from the CE-T1w sequence overlapped to the MNI152, respectively

The spatial variability analysis we conducted in the MNI space provided an error distribution per slice that confirms that ChP volume presents spatial differences between MSegs (Fig. 3a). Axial-wisely, FLAIR was more in agreement than T1w with the reference CE-T1w only in the temporal horn near the head of the hippocampus (MNI152 *z*-coordinate = -12). The T1w sequence was better aligned to the CE-T1w in the other portions of the ChP, while the FLAIR sequence tended to suggest a larger volume in the atrium of the lateral ventricle (MNI 152 *z*-coordinate = 10). In the body of the lateral ventricles, near the fornix (MNI152 *z*-coordinate = 18), a less accurate agreement was observed between non-CE and CE sequences (mean error for T1w > 0.7%; mean error for FLAIR > 1.2%).

Analyzing the ChP coronal-wise, the T1w sequence was in better agreement with the CE-T1w sequence in the majority of the ChP extent. In the atrium of the lateral ventricles (posterior portion, MNI152 *y*-coordinate = -42; anterior portion, MNI152 *y*-coordinate = -35), Fig. 3a shows a great variability of both non-CE sequences. However, the T1w sequence bias was lower than that of FLAIR that exhibited an error of 0.8%. The ChP ends near the anterior part of the fornix (MNI *y*-coordinate = -7), where we measured a comparable error between FLAIR and T1w sequences (error = 0.5%).

Figure 3b shows the frequency maps of the MSegs overlapped to the MNI152 atlas. The frequency map of the CE-T1w MSeg is reported as reference, while for the T1w and FLAIR MSegs, it is reported the difference between the frequency map and the reference. Both axially and coronally, FLAIR sequence tended to overestimate the ChP volume, while the T1w behaves likewise CE-T1w.

## Discussion

To our knowledge, this is the first study that quantitatively evaluates the manual segmentation of the ChP obtained from different sequences, both in terms of intrinsic contrast, overlapping metrics, and spatial variability in a population of multiple sclerosis patients. The aim of our work was to investigate whether the use of CE-T1w sequences, considering the reference standard for ChP imaging, is necessary to quantitatively estimate the ChP volume and which sequence between T1w and FLAIR can be considered the best alternative. Therefore, this investigation might help shedding light on the use of T1-w sequences for ChP segmentation also in other pathologies, rather than MS.

Our purpose started from evidence that suggests reducing the administration of gadolinium-based contrast agents during MRI acquisition due to multiple factors: acute adverse reactions, gadolinium accumulation in the brain [22, 28], the non-eligibility for gadolinium administration for subjects with low glomerular filtration rate (eGFR< 30 mL/min) [29], and the increase in healthcare costs caused by both the contrast itself and the increment in scanning time [30].

We conducted a preliminary inter-rater agreement analysis between a team of a junior and a senior neuroradiologist and a second senior neuroradiologist. We observed that the reproducibility of the segmentation between different operators was very high, and consequently, we used the team segmentations on the entire dataset to conduct further quantitative analysis.

When comparing contrast metrics calculated between ChP and the lateral ventricles, among the available sequences, we expected that CE-T1w and FLAIR could provide better visualization features than T1w sequences (Fig. 1a). CNR and SNR confirms that CE-T1w sequence is the most suitable sequence to visually inspect and depict the ChP and that FLAIR seemed its best alternative. The segmentation metric analysis showed a good agreement between both FLAIR and T1w sequence with the reference CE-T1w. The DSC alone in our case was not sufficient to detect the best candidate to substitute CE-T1w sequences since T1w and FLAIR provided similar DSC values. Nevertheless, more than exploring segmentation overlap, we were interested in the quantification of the ChP volume. ChP volume obtained from the FLAIR sequence was significantly greater (*p* < 0.001) than that obtained with CE-T1w images, as clearly shown in Fig. 2b, while T1w sequences did not provide a specific trend of bias. Moreover, T1w sequences commit lower systematic errors and lower error variability. This finding was also confirmed by Pearson's correlation analysis.

Regarding spatial variability of segmentations in MNI space, FLAIR was more in agreement than T1w with the reference CE-T1w only axial-wise in the temporal horn near the head of the hippocampus, probably due to the greater CNR of the ChP guaranteed by the FLAIR sequence that in this tiny space could potentially improve the segmentation performance. For other portions of the ChP, the intrinsic blurring of the FLAIR image when compared with the T1w brought to an overestimate of the ChP volume.

This study has the two following principal limitations due to its preliminary nature. First, we did not evaluate the intra-rater agreement. Second, the inter-rater agreement was performed on a subset of only ten randomly selected subjects for all available sequences. Despite them, the two rater segmentations were very consistent with high ICC and DSC.

The quantitative analyses we conducted suggest that non-CE T1w sequences might be a candidate as a surrogate of CE-T1w for the MSeg task to estimate ChP volume better than FLAIR sequences. However, when segmenting the ChP, it might be helpful to use both T1w and FLAIR in the anterior portion of the temporal horn of the lateral ventricles. On the contrary, T1w sequences should be preferred in the other ChP portions due to the fixed overestimation bias introduced by the FLAIR. Moreover, the high variability we encountered in several portion of the ChP might highlight the possibility of restricting the ChP volume segmentation, when employing non-CE sequences, to the central part of the ChP, for example excluding regions near the anterior temporal horn or near the fornix, due to the tiny dimension of the ChP that runs parallel to the fornix up to the proximal portion of the anterior horn of the ventricles.

To conclude, the future directions that this study has opened are firstly encouraging the development of automatic tools for the ChP segmentation due to the limitations of the manual segmentation and secondly improving the understanding of the role of the ChP volume in MS, promoting longitudinal studies.

## Data Availability

Data are available from the corresponding author upon reasonable request.

## Abbreviations

|ΔVol%|: Absolute percentage volume difference
CE: Contrast-enhanced
ChP: Choroid plexus
CNR: Contrast-to-noise ratio
DSC: Dice similarity coefficient
FLAIR: T2-weighted fluid attenuated inversion-recovery
ICC: Intraclass correlation coefficient
MNI152: MNI Talairach ICBM 152 2009c nonlinear symmetric template
MSeg: Manual segmentation
SNR: Signal-to-noise ratio
T1w: T1-weighted
ΔVol%: Percentage volume difference
